# *Campylobacter jejuni* colonization and population structure in urban populations of ducks and starlings in New Zealand

**DOI:** 10.1002/mbo3.102

**Published:** 2013-07-22

**Authors:** Vathsala Mohan, Mark Stevenson, Jonathan Marshall, Paul Fearnhead, Barbara R Holland, Grant Hotter, Nigel P French

**Affiliations:** 1 mEpiLabInfectious Disease Research CentreInstitute of Veterinary and Biomedical Sciences, Massey UniversityPalmerston North, New Zealand; 2 EpicentreInstitute of Veterinary and Biomedical Sciences, Massey UniversityPalmerston North, New Zealand; 3 Department of Mathematics and Statistics, Lancaster UniversityLancaster, U.K; 4 School of Mathematics and Physics, University of TasmaniaHobart, Tasmania, Australia; 5 AgResearch, GrasslandsPalmerston North, New Zealand; 6 Allan Wilson Centre for Molecular Ecology and EvolutionPalmerston North, New Zealand; AgResearch, GrasslandsPalmerston North, New Zealand

**Keywords:** *Campylobacter jejuni*, colonization, ducks, population structure, starlings

## Abstract

A repeated cross-sectional study was conducted to determine the prevalence of *Campylobacter* spp. and the population structure of *C. jejuni* in European starlings and ducks cohabiting multiple public access sites in an urban area of New Zealand. The country's geographical isolation and relatively recent history of introduction of wild bird species, including the European starling and mallard duck, create an ideal setting to explore the impact of geographical separation on the population biology of *C. jejuni,* as well as potential public health implications. A total of 716 starling and 720 duck fecal samples were collected and screened for *C. jejuni* over a 12 month period. This study combined molecular genotyping, population genetics and epidemiological modeling and revealed: (i) higher *Campylobacter* spp. isolation in starlings (46%) compared with ducks (30%), but similar isolation of *C. jejuni* in ducks (23%) and starlings (21%), (ii) significant associations between the isolation of *Campylobacter* spp. and host species, sampling location and time of year using logistic regression, (iii) evidence of population differentiation, as indicated by *F*_ST_, and host-genotype association with clonal complexes CC ST-177 and CC ST-682 associated with starlings, and clonal complexes CC ST-1034, CC ST-692, and CC ST-1332 associated with ducks, and (iv) greater genetic diversity and genotype richness in ducks compared with starlings. These findings provide evidence that host-associated genotypes, such as the starling-associated ST-177 and ST-682, represent lineages that were introduced with the host species in the 19th century. The isolation of sequence types associated with human disease in New Zealand indicate that wild ducks and starlings need to be considered as a potential public health risk, particularly in urban areas.

We applied molecular epidemiology and population genetics to obtain insights in to the population structure, host-species relationships, gene flow and evolution of *Campylobacter jejuni* in urban ducks and starlings.

## Introduction

Members of the genus *Campylobacter* are the leading cause of bacterial gastroenteritis in the industrialized world (Newell 2001). *Campylobacter jejuni* and *C. coli*, in particular, are considered to be the most important species causing human disease; *C. jejuni* accounts for ~90% of human cases of campylobacteriosis with *C. coli* accounting for most of the remainder (Lee and Newell 2006). The risk of campylobacteriosis arising from consumption of contaminated food has been extensively studied (Eberhart-Phillips et al. 1997; Samuel et al. 2004; Siemer et al. 2005; Baker et al. 2006, 2007; Miller et al. 2006; Nielsen et al. 2006; Sheppard et al. 2009). Other important modes of transmission include exposure to fecal material from livestock, including ruminants (Savill et al. 2003; Brown et al. 2004; French et al. 2005; Wilson et al. 2008; Carter et al. 2009; Mullner et al. 2009). Although the disease is self limiting it occasionally produces severe sequelae such as Guillain-Barre syndrome (Altekruse et al. 1999). Consumption of contaminated and undercooked meat is generally thought to be the main source of infection (Baker et al. 2006; Galanis 2007; Wilson et al. 2008; Mullner et al. 2009, 2010; Nicol et al. 2010). However, contact with farm animals, pets, and other environmental exposures including wild birds have also been implicated as potential sources of human campylobacteriosis (Friedman et al. 2000; Workman et al. 2005; French et al. 2009).

Multilocus sequence typing (MLST) has become widely used for molecular epidemiological studies of *Campylobacter* spp. (Maiden et al. 1998; Dingle et al. 2001; Maiden 2006). The publicly accessible on-line MLST database enables direct comparison of bacterial strains across the world (Jolley et al. 2001) allowing population-based genetic analyses to be carried out (Maiden 2006). MLST has been extensively used to investigate the population biology of *C. jejuni* in various host species (Maiden 2006; McCarthy et al. 2007). The seven-locus MLST generates sequence types (ST) which can be grouped into clonal complexes (CC) on the basis of the ST sharing four or more alleles in common with a founder genotype (Dingle et al. 2002). Recent studies using MLST have indicated that *Campylobacter* populations vary between host species and environmental niches (McCarthy et al. 2007) and that their lineages are associated with different host sources (Colles et al. 2003, 2008; Fearnhead et al. 2005; Miller et al. 2006). Further, the occurrence of rare allelic profiles, and their distribution among animal sources, has been reported in previous investigations using MLST (Kwan et al. 2008; Carter et al. 2009).

*Campylobacter* spp. have been isolated in a number of birds, both domesticated and wild (Luechtefeld et al. 1980; Fricker and Metcalfe 1984; Frost 2001). The carriage rates among various species of wild birds vary and the differences in carriage rates are thought to be due to factors related to ecology; for example, feeding habits, habitat preferences, and migration patterns (Waldenstrom et al. 2002, 2010; Colles et al. 2009, 2011).

Mallard ducks (*Anas platyrhynchos*) and European starlings (*Sturnus vulgaris*) are potential sources of contamination of water for drinking and recreation purposes, gardens, parks, and children's playgrounds in urban areas, often creating heavy fecal contamination (Odermatt et al. 1998; Colles et al. 2008, 2011). Starlings have been reported to have relatively high carriage rates of *Campylobacter* (40% was identified in the studies performed by Waldenstrom et al. 2002) compared with other wild birds species. Members of CC ST-45, which have been associated with human disease in humans, are also frequently isolated from starlings, wild birds, and livestock (Colles et al. 2003, 2011; Broman et al. 2004; French et al. 2009). However, one recent study showed that none of the *C. jejuni* STs found in wild birds was identified in either domestic animals or humans (Hughes et al. 2009). Nevertheless, Hughes et al. (2009) hypothesized that the livestock-associated strains that were detected in wild bird samples could have arisen from the shared environment that these two species cohabit and that it should be expected that those strains found in wild birds should also be detected in livestock.

Although the proportion of human disease attributable to environmental sources is relatively low, wild birds may still pose a potential risk to human health (Wilson et al. 2008; Sheppard et al. 2009; Colles et al. 2011). From a public health perspective, understanding the relative contributions of environmental exposure pathways is critical for designing appropriate control measures. With this background, the aim of this study was to investigate the prevalence of *C. jejuni* in the feces of starlings and mallard-like ducks found in public access areas in a provincial city in the lower North Island of New Zealand. Hybrids of mallard ducks and gray ducks have been reported in New Zealand (Rhymer et al. 1994), however, based on the phenotypic characteristics and for the purpose of this study we will refer to them as mallard ducks. Further, our aims were to characterize the *C. jejuni* isolates from the above species. We compared the isolates from ducks and starlings in order to provide insights into the population diversity of *C. jejuni* in these species, their host association, and identification of molecular signatures associated with these hosts. A comparison of the *C. jejuni* STs from the sampled wild birds with those disease-causing human genotypes should be helpful when attempting to evaluate the possibility that these species are reservoirs of *C. jejuni* human pathogens in New Zealand. We also compared genotypes isolated from mallard ducks and starlings with the wider population of *Campylobacter* genotypes available in the PubMLST database (http://pubmlst.org/campylobacter/). This comparison provided an overview of the geographical distribution of these STs, the range of hosts in which they are found, and their population structure following their introduction and period of relative isolation in New Zealand. Mallard ducks were introduced into New Zealand in the 18th century from Australia from game stock that originated from England, and European starlings were introduced in the late 19th century (Thomson 1922).

## Experimental Procedures

### Study design

**Figure 1:**
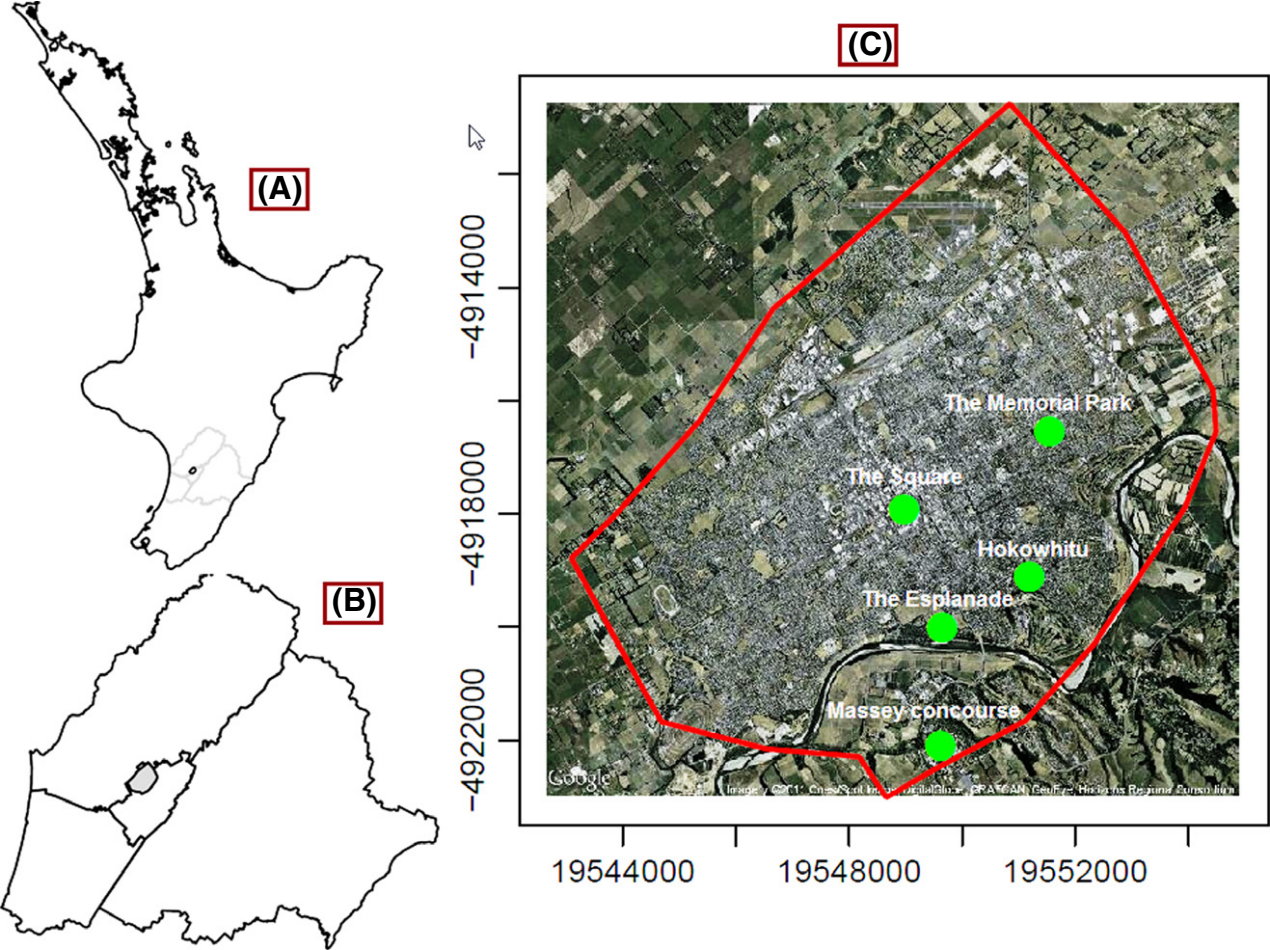
Ducks and starlings sampling sites. The map describes the five sampling sites from where the fecal materials were collected from mallard ducks and starlings for the isolation of *Campylobacter jejuni*. (A) North Island of New Zealand; (B) Manawatu – Palmerston North city; (C) Public parkland sites. The red polygon refers to Palmerston North City.

A repeated cross-sectional study was conducted to determine the prevalence of *Campylobacter* in the feces of mallard ducks (*A. platyrhynchos*) and European starlings (*S. vulgaris*) resident in the city of Palmerston North (longitude 175°, latitude −40°) in the lower North Island of New Zealand. Five public parkland sites within the city limits were selected for sampling: The Square, Hokowhitu, Memorial Park, Massey University and The Esplanade. A children's play area was present in four of the five locations and all sites had at least one duck pond (Fig. 1).

Assuming a prevalence in the order of 35–45% (Colles et al. 2008) calculations were conducted to determine the appropriate number of samples to be 95% certain that our estimate of *C. jejuni* prevalence was within 5% of the true population value. To account for the possibility that prevalence varied across the five sampling sites (i.e., there was clustering of *Campylobacter* within sites) the estimated sample size was multiplied by a design effect of two (Levy and Lemeshow 1999).

### Collection of fecal material

Each of the five study sites was visited at monthly intervals for a period of 17 months, starting in March 2008. Standardization of transport of fecal material and processing for *Campylobacter* isolation was carried out for a trial period of five consecutive months, from March to July 2008 (inclusive). The results reported in this paper are for the period August 2008–July 2009 (inclusive). To facilitate comparison and interpretation of results, the months of sample collection were categorized into warmer times of the year (spring and summer, September 2008–February 2009) and cooler times (autumn and winter, March–July 2009 and August 2008). We classify these two as summer and winter.

At each sampling site fecal material from ducks was collected from areas adjacent to the water sources where ducks gathered. Nesting areas for starlings were identified at each sampling site and fecal samples under each nesting area collected. It is possible that other birds may have defecated under nesting areas, however, during the 5 months trial period, identification of starling fecal material was standardized by waiting and watching the birds defecating and the fecal characteristics were recorded as photographic images. Subsequently, during the main study only fresh fecal material with specific characteristics located directly under starling roosting areas were sampled. Therefore, we are confident that the samples used in this study were from starlings. Fecal samples from both ducks and starlings were collected every month with a sampling interval of 29 days between each sampling round. For ducks, fresh fecal samples (moist) were identified during every sampling occasion to avoid resampling; hence we are certain that a new sample was collected during every sampling round. Each sampling site was divided into four quadrants and three samples from each quadrant collected from both ducks and starlings producing a total of 12 samples for each sampling day. The location of quadrants and the sampling sites did not vary between sampling days. However, ducks and starlings were not constrained to one particular quadrant and/or sampling site. Samples were collected during the early mornings (0600–0800 h) or late evenings (1800–2000 h) on each sampling day.

For the August 2008 sampling round it was not possible to collect a complete set of duck samples from two quadrants at the Hokowhitu site for the following reasons: One quadrant remained unsampled for duck feces as that quadrant was under water and for the second quadrant only two duck samples were identified. Thus, the total number of duck samples for the duration of the study was 716.

### Bacterial isolation and DNA preparation

Fecal samples from all sources were collected simultaneously in transport media (Amies charcoal, Fort Richards, Auckland) and Bolton's enrichment broth (enrichment broth LAB-27.6 G; 50 mL lysed horse blood venous supplies; Antibiotics-LAB-10 mL, Auckland) and transported immediately to the Hopkirk Research Institute Laboratory on the Massey University campus at Palmerston North. Fecal material collected in the transport media was directly streaked onto modified charcoal cefoperazone-deoxycholate (mCCDA) (Fort Richards, Auckland) plates. The inoculated mCCDA plates and Bolton's enrichment broth with fecal material were incubated for 48 h at 42°C in a microaerophilic chamber (MACS VA500 Microaerophilic workstation, Don Whitley Scientific, North Gorsford, Australia). The plates were monitored for growth, and after 48 h the colonies resembling *Campylobacter* spp. were subcultured onto blood agar plates (horse lysed blood agar, Fort Richards). After 48 h the cultures from the Bolton's enrichment broth were swabbed onto mCCDA plates and then the inoculated plates were incubated for another 48 h at 42°C in a microaerophilic chamber. Up to three colonies from the mCCDA plates were subcultured onto horse blood agar plates. The pure colonies isolated from the horse blood agar plates were tested for oxidase reduction (oxidase strips, Fort Richards, Auckland). The colonies that reduced oxidase within 5 sec, as indicated by a purple coloration, were stored in glycerol and processed for DNA isolation. Three colonies of at least 3 mm in diameter were transferred to 1 mL of 2% (weight/volume) Chelex solution in distilled water and boiled at 100°C on heating blocks for 10 min. These were then cooled to room temperature, centrifuged at 13,000 rpm for 10 min, and the supernatants were collected in fresh sterile eppendorf tubes and stored at −20°C.

### Characterization by PCR

Isolates were confirmed to be *Campylobacter* spp. and *C. jejuni* using monoplex polymerase chain reaction (PCR) that targeted 16s rRNA (Linton et al. 1997) for *Campylobacter* spp. and the membrane-associated protein A (*map*A) for *C. jejuni* (Stucki et al. 1995; Mullner et al. 2010), respectively. As our study was primarily focused on characterizing *C. jejuni* from ducks and starlings, species characterization for the non-jejuni campylobacters was not carried out. Genus primer sequences were forward 5' GGATGACACTTTTCGGAGC 3'; reverse 3' CATTGTAGCACGTGTGTC 3'. *Campylobacter jejuni* primer sequences were forward 5' CTTGGCTTGAAATTTGCTTG 3' and reverse 3' GCTTGGTGCGGATTGTAAA 5'. The targets were amplified at 96°C for 2 min for initial denaturation, 96°C for 30 sec, primer annealing at 56°C for 30 sec, and extension at 72_C for 60 sec, for 35 cycles. The PCR reaction mix was comprised of 2 μL 10× PCR buffer (final concentration 1×); 2 μL dNTPs (final concentration 2 mmol/L); magnesium chloride 1 μL, (final concentration 2.5 mmol/L); primers 2 μL each (final concentration 1 mmol/L); Taq DNA polymerase 0.2 μL (final concentration 1 unit per reaction); DNA 2 μL (final concentration 10 ng per μL). The reaction mix was made up to 20 μL with distilled water. The amplicons were examined by agarose gel electrophoresis with results captured using a Bio-Rad gel documentation system (Life Science Group, Canada).

### Multilocus sequence typing

#### Isolate selection for MLST screening and sequencing

Where available, two *C. jejuni* positive isolates were randomly chosen from each sampling site and each species, one from mallard ducks and the other from starlings, for complete MLST characterization to make the study economical. Additional samples were randomly chosen from the sites that had ample *C. jejuni* positive samples to increase the sample size for an analysis of genetic diversity of *C. jejuni*. A total of 121 isolates were characterized but *C. jejuni* was not always isolated from all sampling sites on each occasion (e.g., November 2008–January 2009 for Hokowhitu, December 2008 for The Square, and March 2009 for The Esplanade). MLST was performed as per the method described by Dingle et al. (2001).

#### *fla*A and *por*A typing

Those *C. jejuni* isolates that had complete MLST profiles were further typed by sequencing genes associated with cell surface antigens. The internal fragments in the *fla*A short variable region (SVR) and the internal fragments of *por*A gene were amplified and sequenced for assigning nucleotide allelic numbers (http://pubmlst.org/campylobacter/). The isolates that were difficult to amplify were further processed in the Hopkirk Research Laboratory using primers from the PubMLST database. This was done by optimizing the PCR reaction mix (MgCl_2_ optimized to 2.5 mol/L and primers: 3.2 picomoles) and the PCR program as described in Table S4.

### Statistical analysis

Logistic regression analysis was used to describe the relationship between *Campylobacter* spp. (response variable), and the variables: host, sampling location, and month of sampling. Overdispersion was observed, and thus the model was fit using quasi-likelihood procedure with dispersion parameter estimated from the data using the generalized linear model (GLM) framework of the statistical package (R Development Core Team 2010). A multiplicative interaction term of species and month of sampling was used to assess whether the temporal trend differed between hosts. Species and the month of sampling were used as multiplicative interaction terms in the model to analyze the temporal and the host influences over the response variable, the prevalence of *Campylobacter* spp.

### Genetic analysis

#### Taxonomic richness and diversity

Rarefaction was performed by using the frequency of STs (Gormley et al. 2008) found in ducks and starlings. The frequency distribution of each *C. jejuni* ST was summarized at the sampling sites, species, and seasonal level in an effort to describe how the *C. jejuni* population varied according to sampling sites, species, and time of the year. The analysis was carried out using the contributed R package Vegan (R Development Core Team 2010) that contains a rarefaction function. In rarefaction analysis, the horizontal axis of the plot represents the number of samples used for analysis and the vertical represents the diversity or the number of genotypes identified in the specified number of samples. Diversity indices such as Simpson index 1-D and Shannon index were measured using PAST (Hammer et al. 2009) to analyze the *C. jejuni* population in ducks and starlings. Simpson index 1-D measures the evenness of the community and has a scale from 0 to 1, where 0 indicates that all taxa are equally present and 1 indicates that one taxon dominates the community completely. Whereas the Shannon index takes the number of individuals as well as number of taxa into account and varies from 0 for communities in which a single taxon dominates to values above 0 for communities with many taxa occurring at a similar frequency.

#### Population differentiation: AMOVA and *F*_ST_

Analysis of molecular variance (AMOVA) was performed to compare the effect of sampling site, host species, and sampling period on the population differentiation and population structure of C. *jejuni* using Arlequin v3.11 (Excoffier et al. 2005). Analysis at different levels, referred to as “hierarchical analysis” was carried out using AMOVA which divides the total variance into different covariance components such as within population, within groups among populations, and interpopulation differences (Excoffier et al. 2005). While analyzing the genetic structure for different hierarchical levels, three hierarchical F-statistics are derived known as the fixation indices (expressed as components of AMOVA). The fixation indices include: *F*_ST_, a fixation index that measures the variance among subpopulations relative to the total variance, *F*_SC_ that measures variance among subpopulations within groups, and *F*_CT_, the variance among groups relative to the total variance. *F*_ST_ quantifies the genetic differentiation among populations under comparison using an index ranging from 0 to 1. A zero value implies that there is no differentiation between populations and a value of one implies the two populations are completely separate Wright 1978; Wright 1984). Wright (1978) has further suggested the qualitative guidelines for the interpretation of *F*_ST_ such as: (1) the range 0.0–0.05 may be considered as indicating small or limited genetic differentiation; (2) the range 0.05–0.15 indicates moderate genetic differentiation, (3) the range 0.15–0.25 indicates great genetic differentiation, and (4) the values of *F*_ST_ above 0.25 indicate very great genetic differentiation. The genetic distance of *C. jejuni* populations found in ducks and starlings at different time periods and at different sampling sites were calculated using MLST allelic profiles. In turn, all these fixation indices facilitate inference on the gene flow between populations compared.

Phylogenetic relationships among the STs were analyzed using Bionumerics v6.1.4. A minimum spanning tree (MST) was constructed using the allelic profile data set for ducks and starlings collected from March 2008 (the trial period included) to July 2009. MST is an alternative approach to show the relationships among isolates from bacterial populations (Prim 1957). A MST links the genotypes or STs that are closely related within their lineages or CCs, identifying the most likely “ancestral” genotype of each CC. The ancestral genotypes are called the “consensus” clones and the radial spread of STs from the consensus clones are reflected by a series of circles and the size of the circles represent the number of isolates per ST (Prim 1957).

## Results

### Prevalence of *Campylobacter* spp. and *C. jejuni*

**Figure 2:**
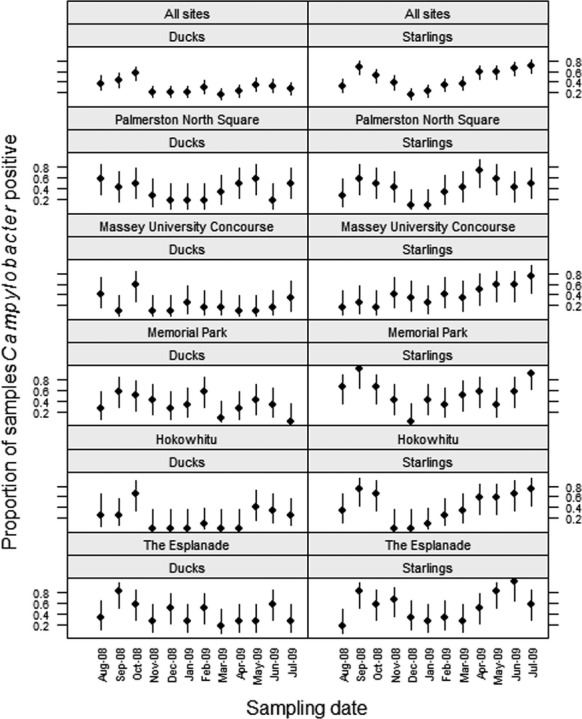
Prevalence of *Campylobacter* by sampling date and sampling site. The graph describes the prevalence of *Campylobacter* in the feces of mallard-like ducks and starlings from August 2008 to July 2009 in each sampling site. The vertical lines in the figure refer to 95% confidence intervals.

The overall prevalence of *Campylobacter* spp. in the sampled wild bird feces was 38% (95% CI 35–40%; 539 of 1436). The prevalence of C*ampylobacter* spp. in starlings (46%, 95% CI 42–50%) was significantly higher than that of ducks (30%, 95% CI 26–33%). Further, the prevalence of *Campylobacter* spp. varied by month and site as shown in Figure 2 and the model results are shown in Table 1. There was a trend of relatively high prevalence during the spring months of September and October, and the winter months of May, June, and July and low prevalence during the summer. The inclusion of month in the logistic regression model was highly significant, with the months of September, October, May, June, and July estimated to be significantly different (*P* < 0.001) compared to the baseline month of December 2008 (Table 1). When a multiplicative interaction term for species and month was introduced into the model, it was significant (*P* value 0.022), however, differences in the relationship between *Campylobacter* spp. and month for starlings and ducks were subtle, and could mainly be attributed to a higher prevalence in starlings in the months of April and July. There were also significant differences between sites; the Esplanade sampling site showed the highest prevalence of *Campylobacter* spp. (Table 1).

**Table:** The relationship between prevalence of *Campylobacter* spp. and *C. jejuni* in ducks and starlings and the variables month of year and sampling location

Variable	*Campylobacter* spp.				*C. jejuni*						
	Coefficient	SE	*P* value	Coefficient	SE	*P* value
Intercept	−1.57	0.32	<0.0001	−1.71	0.44	NS
Species			<0.001^1^			NS^1^
Duck	Ref			Ref		
Starling	0.42	0.13	<0.001	−0.13	0.18	NS
Site			<0.001^1^			<0.001^1^
Esplanade	Ref			Ref		
Hokowhitu	−0.62	0.2	<0.003	−0.64	0.3	<0.03
Memorial Park	−0.09	0.19	NS	−0.02	0.27	NS
Massey	−0.6	0.2	<0.004	−0.67	0.3	<0.02
Square	−0.21	0.2	NS	−0.1	0.28	NS
Month			<0.00001^1^						<0.00001^1^
August-2008	0.73	0.31	<0.05	0.59	0.52	NS
September-2008	1.58	0.3	<0.001	1.72	0.48	<0.001
October-2008	1.56	0.3	<0.001	1.68	0.48	<0.001
November-2008	0.57	0.31	NS	0.54	0.52	NS
December-2008	Ref			Ref		
January-2009	0.24	0.33	NS	0.42	0.53	NS
February-2009	0.69	0.31	<0.05	0.59	0.52	NS
March-2009	0.41	0.32	NS	0.15	0.55	NS
April-2009	0.92	0.3	<0.01	0.7	0.51	NS
May-2009	1.14	0.3	<0.001	0.85	0.5	<0.05
June-2009	1.12	0.29	<0.001	0.75	0.51	<0.05
July-2009	1.06	0.29	<0.001	0.36	0.53	NS

Dispersion parameter for quasibinomial family for genus was taken to be 1.38. Dispersion parameter for quasibinomial family for species was taken to be 1.92. Ref, reference category; NS, not significant; SE, standard error.

1 *P* value based on likelihood ratio test statistic for variable as a whole.

Three hundred and fifteen *C. jejuni* isolates were recovered from 1436 fecal samples from mallard ducks and starlings giving an overall estimated *C. jejuni* prevalence of 22% (95% CI 20–24%). The logistic regression model which considered *C. jejuni* as an outcome (Table 1) showed that the prevalence of *C. jejuni* varied significantly by month (*P* < 0.0001) and site (*P* < 0.001), but there was no evidence of a significant difference between starlings and ducks. The prevalence of *C. jejuni* during the spring months of September and October and the winter months of May and June was significantly higher than the month of December (Table 1). Similarly, a multiplicative interaction term inclusion for species and month into the model was significant (*P* value 0.03). However, the observed differences in the relationship between *C. jejuni* prevalence and the month were mainly due to a higher prevalence in starlings during the months of September and October in summer and May and June in winter.

### Genotype richness and diversity by host

Forty three different STs were obtained from 121 isolates from ducks and starlings and these were assigned to 11 CC. CC ST-45 was the most predominant ST found in the two species accounting for 23% (28 of 121) of isolates.

The most common CC in mallard ducks was CC ST-1034, accounting for 28% (18 of 64) of samples. The second largest clonal complex was CC ST-45, accounting for 19% (12 of 64) of samples, followed by CC ST-692 which accounted for 9% (six of 64) of samples. Twenty two of the 64 *C. jejuni* STs from ducks could not be assigned to a clonal complex.

The majority of the starling isolates were assigned to CC ST-45, accounting for 28% (16 of 57) of starling isolates, and the next largest was CC ST-177 (9%, five of 57). Twenty three isolates could not be assigned to a CC. Other CCs were ST-677, ST-682, ST-692, ST-1034, and ST-42 each accounting for 4% (two of 57 isolates) and ST-21, ST-1304, and ST-1332 accounted for 2% (one of 57 isolates) each. The STs that were present both in ducks and starling were ST-1324, ST-1342, ST-137, ST-2378, ST-3961, ST-45, ST-53, ST-583, ST-692, ST-991, and ST-992 although the frequency of each ST varied between the two species.

**Figure 3:**
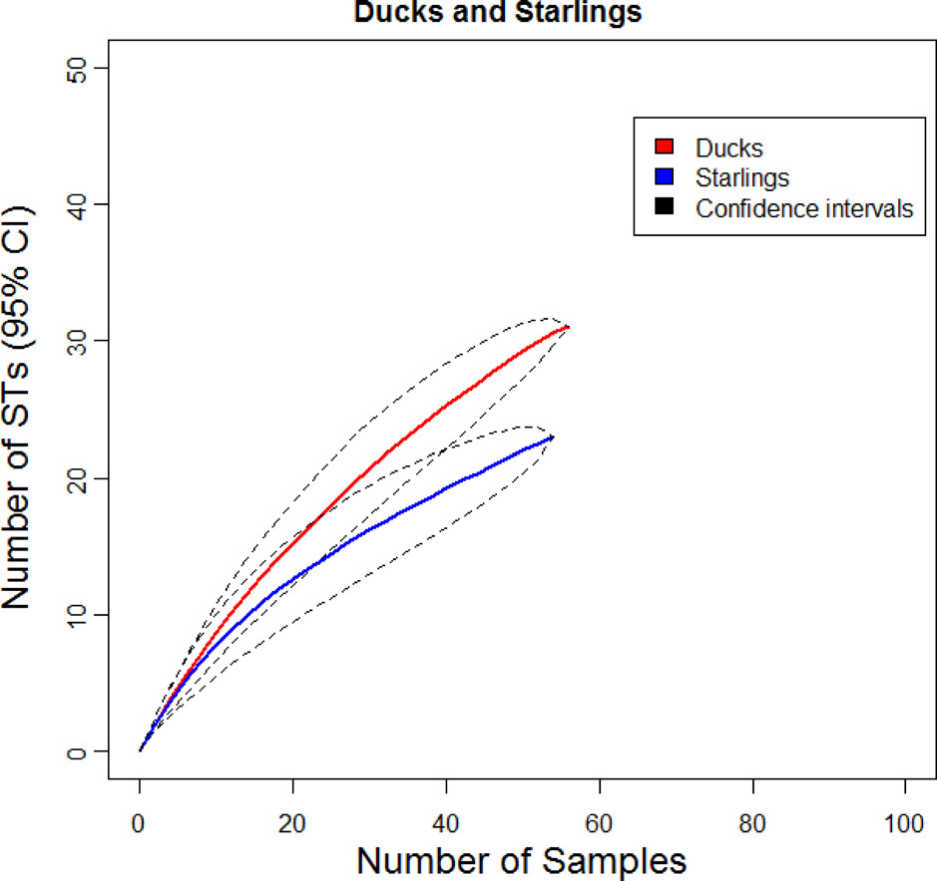
Rarefaction curves of *Campylobacter jejuni* sequence types (ST) isolated from ducks and starlings. The *X-*axis denotes the number of samples genotyped and the *Y-*axis denotes the number of different STs present in the given number of isolates. The population of *C. jejuni* genotypes in ducks is taxonomically richer compared with starlings.

Rarefaction curves showing the taxonomic richness of STs as a function of the number of isolates for each host are shown in Figure 3. A slope of zero in the rarefaction curves indicate that the maximum genetic diversity has been reached and that it is unlikely that more genetic diversity will be identified if more samples are analyzed. The rarefaction curve for *C. jejuni* genotypes indicates greater genotype richness in ducks compared with starlings in the samples analyzed and that further sampling would result in the identification of more genotypes.

**Figure 4:**
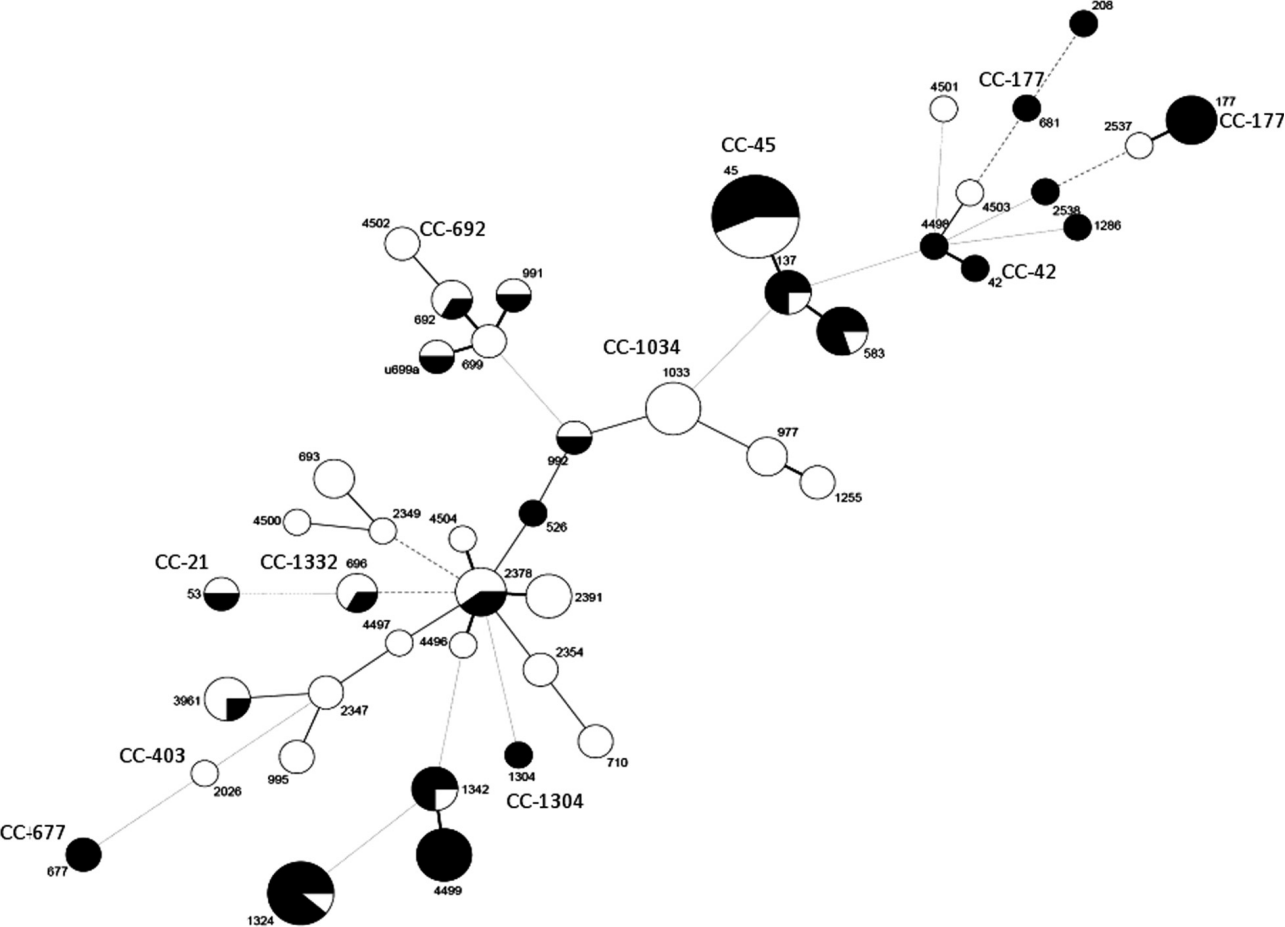
Minimum spanning tree (MST) of *Campylobacter jejuni* sequence types (ST) from mallard ducks and starlings. Each node represents a sequence type, and the size of the circle is proportional to the number of isolates. The proportion of genotypes isolated from ducks (white) and starlings (black) are shown as a pie chart in each node.

A MST was constructed to display the relatedness among the *C. jejuni* STs isolated from the ducks and starlings from the five sampling sites (Fig. 4). This figure shows the relationship between STs that are either shared between the two host species, or only isolated in one of the host species. The point estimates of Simpson and Shannon indices of diversity were greater in ducks (0.95, CI 0.92–0.96 and 3.30, CI 2.84–3.4 respectively) compared to starlings (0.92, CI 0.91–0.95 and 2.80, CI 2.72–3.30 respectively), which is consistent with the rarefaction analysis, but the bootstrapped confidence intervals overlapped.

### Genotype diversity by sampling period

The distribution of ST and CC in summer and winter are described for ducks and starlings in Tables S3–S6. In ducks the most commonly isolated STs were either unassigned to a CC or were members CC ST-1034 in both summer and winter. In starlings, STs belonging to unassigned CCs were common in both seasons, whereas members of CC ST-45 were isolated more frequently in summer (13/28 samples) compared to winter (3/29 samples).

### Analysis of molecular variance and *F*_ST_

*F*_ST_, the genetic fixation index, was used to quantify the extent of genetic differentiation among populations. The index ranges from 0 to 1; a value of 0 indicates no differentiation between populations while a value of 1 indicates the populations are completely different or separate (Wright 1984).

Pairwise *F*_ST_s were calculated using a hierarchical model (AMOVA) to estimate the components of variation, comparing the genetic differentiation of *C. jejuni* populations between ducks and starlings from each sampling site and at different sampling periods.

**Figure 5:**
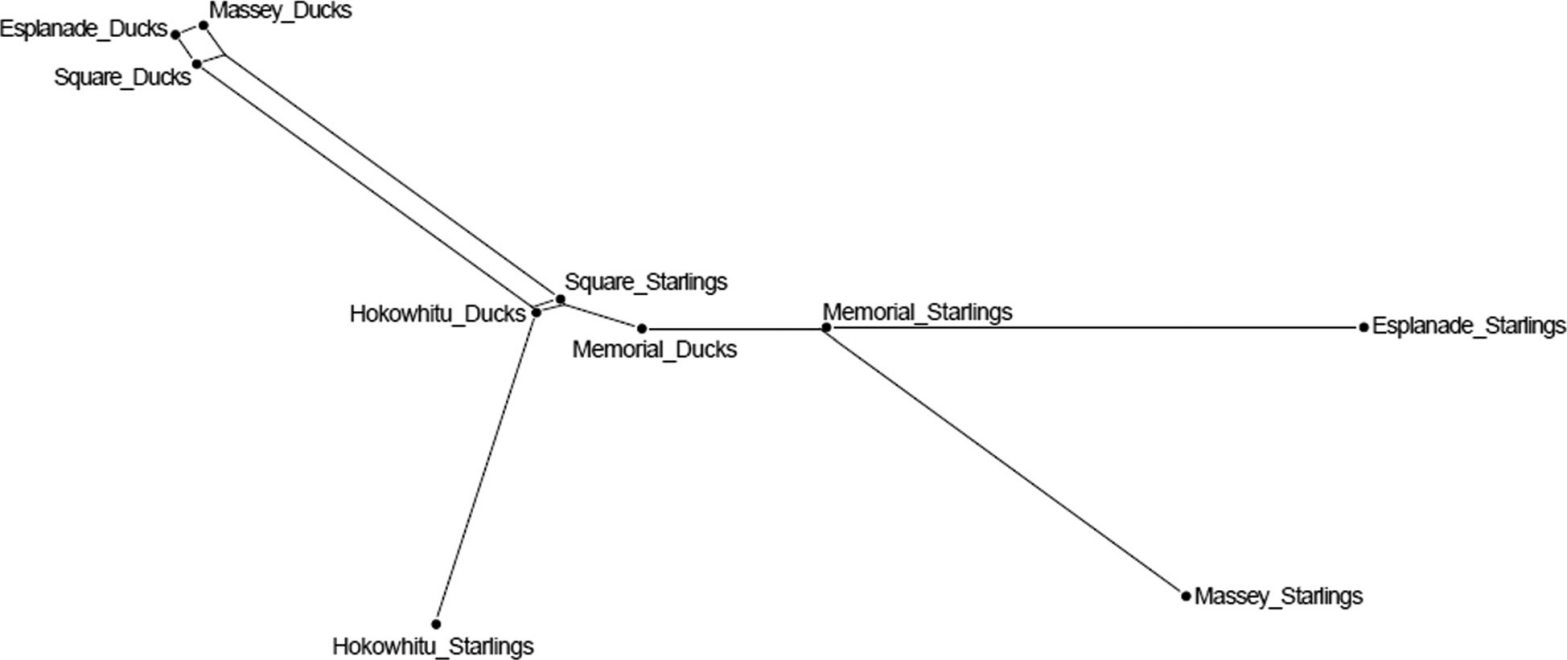
Population differentiation NeighborNet tree constructed using SplitsTree based on *F*_ST_ values. The differentiation of *Campylobacter jejuni* populations from the ducks and starlings from five sampling sites is described in this plot. Further details of the *F*_ST_ and *P* values are provided in the supplementary material.

The overall *F*_ST_ value between ducks and starlings was 0.08 (*P* < 0.0001), which indicates ~8% of the molecular variance was attributable to host species; representing a small but highly significant degree of differentiation between the *C. jejuni* populations of ducks and starlings. The *C. jejuni* populations in ducks, particularly in the Esplanade, Massey and Square sites, display a low degree of differentiation, whereas the *C. jejuni* isolated from starlings are more differentiated from each other across sites and from the *C. jejuni* isolated from ducks (Fig. 5). Figure 5 represents the *F*_ST_ – population differentiation as a NeighborNet tree, whereby the distance between nodes represents the degree of differentiation at the population level between host species at different sites. Although AMOVA showed some evidence of differentiation between ducks and starlings, there was no statistical evidence of differentiation between sites; within hosts (*F*_ST_ values were very small). The overall *F*_ST_ value for the comparison of summer and winter (*F*_ST_ = 0.06; *P* = 0.001 for ducks and *F*_ST_ = 0.08; *P* = 0.002 for starlings) was indicative of seasonal population differentiation in both host species.

### *Campylobacter jejuni* cell surface antigens typing and diversity

All the 121 MLST characterized isolates were subjected to further typing by sequencing the genes associated with cell surface antigens, however, despite repeated processing and several amplification attempts 17 *por*A alleles and 21 *fla*A alleles from both ducks and starlings could not be sequenced. A total of 32 different *por*A alleles were characterized from 101 MLST characterized *C. jejuni* isolates. *por*A allele 6 was the predominant allele in ducks (seven of 40). *por*A alleles 970 and 44 were found to be predominant among starlings. The duck *fla*A nucleotide alleles were more diverse than those alleles from starlings. The *fla*A nucleotide alleles 1170, 1219, 1221, 1222, 1235, 16, 209, 213, 219, 520, 56, 69, 73, 787, 85, and 89 were found only in ducks. The MLST allelic profiles, *fla*A and *por*A allelic numbers of *C. jejuni* isolates of ducks and starlings are summarized in Tables S1, S2.

### Comparison with wider population of genotypes in the PubMLST database

The *C. jejuni* ST populations were compared with global *C. jejuni* ST populations available in the PubMLST database. Of 43 *C. jejuni* STs, ten (ST-2538, ST-3961, ST-4496, ST-4497, ST-4498, ST-4499, ST-4500, ST-4501, ST-4502, ST-4503, ST-4504) were first described in this study. In the United Kingdom ST-1033 has been predominantly isolated from geese, whereas in our study this ST was highly prevalent in ducks (5% of the total number of isolates). ST-177 was prevalent only in starling fecal material in our study which is in agreement with previous New Zealand studies and reports from the rest of the world which referred to this complex as a starling adapted complex (Colles et al. 2008; French et al. 2009; Hughes et al. 2009). Further, this ST has been isolated from starlings and bathing beaches in the United Kingdom in large numbers and in Spain from environmental water. ST-692, ST-693, ST-696, ST-699, and ST-710 were reported predominantly from geese in the United Kingdom with an exception of ST-696 which was also isolated from cattle and a turkey in Germany while these STs were isolated from both ducks and starlings in our study. The results of comparison of STs from our study with the global population of *C. jejuni* STs are tabulated in Table 2 to provide further information on STs and the host species from which they were isolated worldwide.

**Table:** Sequence types (STs) found in ducks and starlings in Palmerston North, New Zealand, compared with the wider population of genotypes in the PubMLST database

ST	Species	Chicken	Turkey	Goose	Starling	Duck	Wild bird	Sheep	Cattle	Pig	Pets	Env_ water	Human
42	S	18	−	−	1	−	3	11	29	−	−	−	121
45	DS	184	12	−	−	−	9	4	21	−	3	65	417
53	DS	31	−	−	2	−	1	1	3	−	−	−	390
137	DS	25	−	7	1	−	−	2	−	−	−	8	121
177	S	–	−	−	48	−	−	−	−	−	−	4	2
208	S	−	−	−	−	−	−	−	−	−	−	1	−
526	S	−	−	−	−	−	−	−	−	−	−	−	1
583	DS	19	−	−	−	−	2	−	2	−	−	2	51
677	S	5	−	−	2	−	6	−	1	−	−	3	24
681	S	−	−	−	−	−	−	−	−	−	−	−	−
692	DS	−	−	5	−	−	6	−	−	−	−	−	−
693	D	−	−	11	−	−	6	−	−	−	−	−	−
696	S	−	1	10	−	−	2	−	1	−	−	−	4
699	D	−	−	8	−	−	2	−	−	−	−	−	−
710	D	−	−	14	−	−	3	−	−	−	−	−	1
991	DS	−	−	−	−	−	13	−	−	−	−	1	1
992	DS	−	−	−	−	−	4	−	−	−	−	2	−
995	D	−	−	−	−	−	3	−	−	−	−	−	−
1033	D	−	−	16	−	−	−	−	−	−	−	−	−
1255	D	−	−	1	−	−	15	−	−	−	−	−	1
1286	S	−	−	−	−	−	4	−	−	−	−	−	−
1304	S	−	−	−	−	−	11	−	−	−	−	−	−
1324	DS	−	−	−	−	−	7	−	−	−	−	−	−
1342	DS	−	−	−	−	−	15	−	−	−	−	−	−
2026	D	−	−	−	−	−	−	−	−	−	−	−	1
2347	D	−	−	−	−	−	−	−	−	−	−	1^1^	−
2349	D	−	−	−	−	1^1^	−	−	−	−	−	−	−
2354	D	−	−	−	−	1^1^	−	−	−	−	−	−	−
2378	DS	1^1^	−	−	−	−	−	−	−	−	−	−	−
2391	D	−	−	−	−	−	−	−	−	−	−	1^1^	−
2537	D	−	−	−	−	−	1^1^	−	−	−	−	−	−
2538	S	−	−	−	−	−	1^1^	−	−	−	−	−	−
3961	DS	−	−	−	−	−	3^2^	−	−	−	−	−	−
4496	D	−	−	−	−	1^2^	−	−	−	−	−	−	−
4497	D	−	−	−	−	1^2^	−	−	−	−	−	−	−
4498	S	−	−	−	1^2^	−	−	−	−	−	−	−	−
4499	S	−	−	−	6^2^	−	−	−	−	−	−	−	−
4500	D	−	−	−	1^2^	1^2^	−	−	−	−	−	−	−
4501	D	−	−	−	1^2^	1^2^	−	−	−	−	−	−	−
4502	D	−	−	−	2^2^	1^2^	−	−	−	−	−	−	−
4503	D	−	−	−	1^2^	1^2^	−	−	−	−	−	−	−
4504	D	−	−	−	1^2^	1^2^	−	−	−	−	−	−	−

DS, ducks and starlings.

1 Isolated in New Zealand.

2 Isolated in the present study and submitted to the PubMLST database.

Several of the STs isolated from ducks and starlings in this study have been implicated as a cause of disease in humans both in New Zealand and overseas. ST-1255, ST-137, ST-696, ST-677, ST-583, ST-526, ST-42, ST-45, ST-53, and ST-2026 have accounted for numerous sporadic human campylobacteriosis cases in countries like the United Kingdom, Scotland, Germany, Belgium, England, The Netherlands, Australia, and Canada (http://pubmlst.org/campylobacter/).

## Discussion

Most of the studies on *Campylobacter* conducted in New Zealand have focused on food sources and reports on wild birds are very scarce. Considering that New Zealand until recently has ranked highest in human campylobacteriosis notification rates (Baker et al. 2007; Marler 2010), a better understanding of distribution of *Campylobacter* spp. in different host species and transmission is essential for policy development and implementing control measures. The primary aim of this study was to estimate the prevalence of *Campylobacter* spp. and *C. jejuni* in ducks and starlings at different time periods and sites. The secondary aim was to analyze the population structure of *C. jejuni* and to determine the population differentiation for different host species at different sampling sites and at different sampling periods. This is of particular interest given the history of introduction of wild birds into New Zealand by European settlers, and its relative geographical isolation. The sites investigated in our study have been identified as major recreational spots that are commonly accessible with a high potential for human interaction with ducks and other urban bird species. Starlings and ducks were chosen as they were the most commonly interacted species in these sites which in turn may represent a source of exposure for *Campylobacter* for the human population in Palmerston North.

The overall prevalence of *Campylobacter* spp. was estimated to be 37% (95% CI 35–40%; 542 of 1458) which is relatively high compared with estimates for migrating birds (22%) and relatively low compared with estimates for aquatic birds feeding on invertebrates (50%) as reported by Waldenstrom et al. (2002). In the European starlings in this study, the estimated prevalence of *Campylobacter* spp. (48%) was relatively high compared with previous prevalence estimates of 33% to 40% (Waldenstrom et al. 2002; Colles et al. 2008; Hughes et al. 2009). It should be noted, however, that the prevalence of *Campylobacter* spp. in starlings is generally higher compared with other avian species (Colles et al. 2008; Hughes et al. 2009). Importantly, these estimates are likely to be an underestimate due to imperfect test sensitivity – we selected three colonies per sample for further testing and it is likely that if more had been examined the sensitivity and estimated prevalence would have increased.

In contrast, the prevalence of *C. jejuni* in ducks (23%) and starlings (21%) were relatively low compared with previous prevalence estimates in Anatidae (ducks, 30.6%) and European starlings (29.9%) (Waldenstrom et al. 2002; Brown et al. 2004; Colles et al. 2008). Differences in these prevalence estimates may be due to several factors such as the risk of becoming colonized by the bacteria, sampling procedures, sample size, and sensitivity of culture techniques. Further, differences may also arise due to the type of samples examined, for example, intestinal samples have been found to give a higher isolation rate compared with fecal samples (Stanley et al. 1998). The apparent bimodal seasonal prevalence in *Campylobacter* spp. and *C. jejuni* in ducks and starlings, with peaks in spring and late autumn/winter has not been previously reported. The early spring peak in prevalence in ducks was in agreement with one recent study (Colles et al. 2009), although that particular study estimated *Campylobacter* spp. prevalence in swan and geese (not ducks). Few other studies (Waldenstrom et al. 2002; Broman et al. 2004) identified a greater level of shedding of *Campylobacter* spp. in the autumn. There was marked seasonality in the prevalence of *C. jejuni* STs both in ducks and starlings.

In general, the observation of high prevalence of the ST-45 complex in spring and early summer was consistent with other reports describing seasonal prevalence of this CC in other animal sources and human campylobacteriosis cases (Colles et al. 2003, 2008, 2009; Sopwith et al. 2008; Grove-White et al. 2011). Reports of seasonality of *C. jejuni* populations amongst wild birds are few and are limited to a small number of studies (Colles et al. 2008, 2009). In our study the majority of STs from ducks (*n* = 22) and starlings (*n* = 21) remained unassigned which was in agreement with those identified in previous studies (Colles et al. 2008; French et al. 2009; Hughes et al. 2009).

Rarefaction analysis of the MLST data revealed higher genetic diversity within the *C. jejuni* isolates from ducks compared to starlings, although the confidence intervals for other measures of diversity overlapped. We hypothesize that the increased diversity and the high prevalence of the unassigned STs may be due to the flocking behavior of birds at different times of the year. Aggregation of birds from different areas is thought to result in mixing of different strains of bacteria from different geographical locations. It may be hypothesized that the physiological changes that occur in the avian gut at different times of the year (related to, e.g., diet and feeding habits) would alter the composition of the gut microbiota which, in turn, could influence the overall genetic diversity of *C. jejuni* in these species.

Although the host-genotype associations have been examined in a number of studies, most have focused on food animals and only a few have investigated wild bird species (McCarthy et al. 2007; Colles et al. 2008, 2009, 2011; French et al. 2009). Further, the unique history of species introductions, including wild birds, and the relative geographical isolation of New Zealand, provide an ideal setting to explore the population biology of *C. jejuni*. Our study provided evidence of similar host-genotype association for *C. jejuni* STs as reported in other parts of the world, but with some differences. For example, the isolation ST-177 in starlings was consistent with previous reports of this being a starling-associated genotype (Colles et al. 2008, 2009; French et al. 2009), that may have been introduced into New Zealand in starlings imported by the “Acclimatisation Societies” in the late 19th century (Thomson 1922). In contrast, interrogation of the *Campylobacter* MLST database and previous reports (Colles et al. 2008) showed that the ST-1034, ST-692, and ST-1332 complexes have only been isolated from geese in other countries, whereas in our study these were predominantly isolated from ducks. In addition, two isolates from the ST-1034 complex and one isolate from the ST-1332 complex were isolated from European starlings in the present study, which may indicate localized interspecies transmission.

There is some controversy about host association between *C. jejuni* genotypes and wild birds (Hughes et al. 2009), with some studies questioning the association between *C. jejuni* genotypes and wild birds (Colles et al. 2003) and others providing evidence of association (Colles et al. 2008, 2009). French et al. (2009) raised a possibility that, the resemblance between the CC found in New Zealand and the United Kingdom is suggestive of a common ancestor that originated from introduced European birds. This present study lends support to this as the majority of the genotypes isolated in this study are also found in Europe. The heterogeneity in the *C. jejuni* populations and the diversity in the cell surface antigens in ducks compared to starlings may be due to the aquatic feeding habits of the ducks and the possibility that ponds could contain a variety of contaminants including the feces of nonaquatic birds. Further, coinfection with different strains and/or species of *Campylobacter* and/or different species may lead to recombination between genes encoding for cell surface antigens, rendering *C. jejuni* genotypes with high virulence (Nuijten et al. 1990, 2000; Wassenaar et al. 1995; Harrington et al. 1997).

There was evidence of intersite transmission and there was significant host association. Particularly during the summer, *C. jejuni* STs from starlings that are associated with human infection (ST-45, ST-137, ST-583, ST-677) were found in all sampling sites (data not shown), which implies that these sites could be a potential source of infection for humans. Furthermore, ST-45 has been found to be strongly associated with the early summer seasonal peak of human campylobacteriosis and is well recognized to colonize domestic poultry and to survive outside the host compared with other STs (Sopwith et al. 2006, 2008; Kairenlampi et al. 2007; French et al. 2009; Habib et al. 2010). Early summer seasonal peak in human campylobacteriosis in New Zealand may be partly due to direct environmental exposures as well as the already established food-borne pathways.

In conclusion, this study has examined the prevalence, population structure, genetic diversity, and host-genotype association of *Campylobacter* spp. in ducks and starlings, in an urban area of New Zealand. The combined approach of genotyping, population genetics, and epidemiological modeling has enabled us to characterize the distribution and potential transmission of *C. jejuni* lineages in ducks and starlings at different sampling sites and time periods. Although our study has identified a similar trend of host-genotype association in ducks and starlings as reported previously, it has also identified subtle differences in these patterns. Further, these observed variations reflect genetic exchange between *C. jejuni* and the potential impact of translocation and mixing of different species of mammals or birds on evolution. Finally, this study has important implications for the management of *C. jejuni* contamination of the recreation spots and/or the environment, and associated public health risks.

## Supporting Information

**Figure S1.** Prevalence of *Campylobacter jejuni* in mallard ducks and starlings. The graph describes the prevalence of *C. jejuni* in the fecal materials of mallard ducks and starlings during each sampling occasion and in each sampling site. CI in the figure refers to confidence interval. **Table S1. **MLST allelic profiles, *fla*A, *por*A nucleotide alleles and their frequency in ducks. **Table S2. **MLST allelic profiles, *fla*A, *por*A nucleotide alleles and their frequency in starlings. **Table S3.** Clonal complexes, sequence types and their prevalence during summer in ducks (The months of spring and summer; September 2008–February 2009 were categorised as summer as these months are warmer during the year). **Table S4.** Clonal complexes, sequence types and their prevalence during winter in ducks (The months of autumn and winter; March–July 2009 and August 2008 were categorised as winter as these months are cooler during the year). **Table S5.** Clonal complexes, sequence types and their prevalence during summer in starlings. **Table S6.** Clonal complexes, sequence types and their prevalence during winter in starlings. **Table S7.** Analysis of molecular variance (AMOVA) Population pairwise *F*_ST_s for ducks and starlings at different sampling sites. **Table S8. ***fla*A and *por*A typing.
