# Supplementary material for: Campylobacter jejuni colonization and population structure in urban populations of ducks and starlings in New Zealand
Source: Microbiologyopen. 2013 Jul 22;2(4):659–73. doi: 10.1002/mbo3.102 (PMC3831628; doi:10.1002/mbo3.102)
Supplement: Supplementary file 1 — Figure S1. Prevalence of Campylobacter jejuni in mallard ducks and starlings. The graph describes the prevalence of C. jejuni in the fecal materials of mallard ducks and starlings during each sampling occasion and in each sampling site. CI in the figure refers to confidence interval. Table S1. MLST allelic profiles, flaA, porA nucleotide alleles and their frequency in ducks. Table S2. MLST allelic profiles, flaA, porA nucleotide alleles and their frequency in starlings. Table S3. Clonal complexes, sequence types and their prevalence during summer in ducks (The months of spring and summer; September 2008–February 2009 were categorised as summer as these months are warmer during the year). Table S4. Clonal complexes, sequence types and their prevalence during winter in ducks (The months of autumn and winter; March–July 2009 and August 2008 were categorised as winter as these months are cooler during the year). Table S5. Clonal complexes, sequence types and their prevalence during summer in starlings. Table S6. Clonal complexes, sequence types and their prevalence during winter in starlings. Table S7. Analysis of molecular variance (AMOVA) Population pairwise FSTs for ducks and starlings at different sampling sites. Table S8. flaA and porA typing. [file mbo30002-0659-SD1.docx]

*Campylobacter jejuni* colonisation and population structure in urban populations of ducks and starlings in New Zealand

V. Mohan^1^, M. Stevenson^2^, J. Marshall^1^, P. Fearnhead^5^, B. Holland^6^, G. Hotter^4^ and N. P. French^1,3*^

1 - ^m^EpiLab, Infectious Disease Research Centre, Institute of Veterinary and Biomedical Sciences, Massey University, Palmerston North, New Zealand

2 - Epicentre, Institute of Veterinary and Biomedical Sciences, Massey University;

3 - Allan Wilson Centre for Molecular Ecology and Evolution

4 - AgResearch, Grasslands, Palmerston North, New Zealand

5 - Department of Mathematics and Statistics, Lancaster University, Lancaster, United Kingdom

6 - Department of Mathematics, Theoretical Phylogenetics Group, University of Tasmania, Australia.

*- Corresponding author. Contact address:N.P.French@massey.ac.nz

**Supplementary material**

Summary:

This supplementary material provides further information on the details described in the manuscript. This section gives an additional description of the laboratory procedures performed in this study. Although the results and the patterns of population differentiation were presented and discussed in the manuscript, further details of *C. jejuni* prevalence and tables of Fst values and the p-values are presented in this supplementary material.

**Results:**

***C. jejuni* prevalence:**

Figure SF1


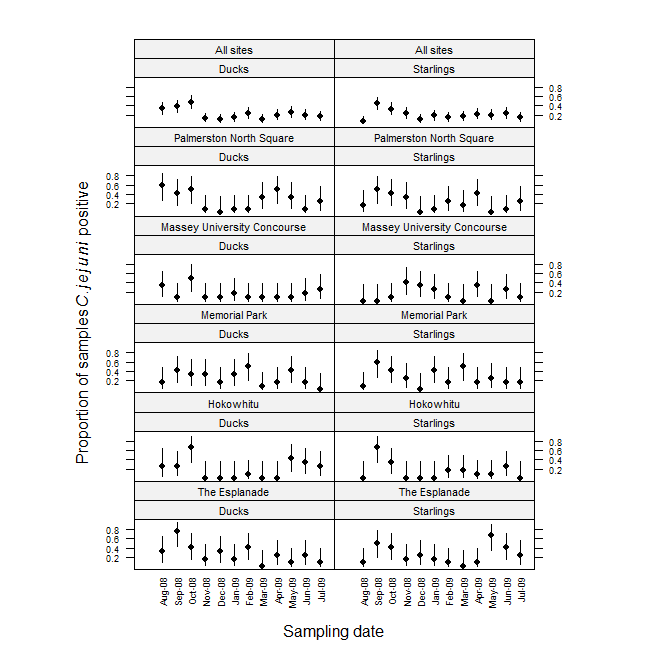


Figure SF1: Prevalence of *C. jejuni* in mallard ducks and starlings. The graph describes the prevalence of *C. jejuni* in the faecal materials of mallard ducks and starlings during each sampling occasion and in each sampling site. CI in the figure refers to confidence interval.

Table ST 1. MLST allelic profiles, *fla*A, *por*A nucleotide alleles and their frequency in

ducks.

| Frequency | flaA | porA | ST | CC | aspA | glnA | gltA | glyA | pgm | tkt | unc |
| --- | --- | --- | --- | --- | --- | --- | --- | --- | --- | --- | --- |
| 1 | 32 | 27 | 53 | 21 | 2 | 1 | 21 | 3 | 2 | 1 | 5 |
| 1 | X | X | 4503 | 42 | 1 | 2 | 9 | 5 | 5 | 9 | 21 |
| 3 | 21 | 44 | 45 | 45 | 4 | 7 | 10 | 4 | 1 | 7 | 1 |
| 1 | 85 | 53 | 137 | 45 | 4 | 7 | 10 | 4 | 42 | 7 | 1 |
| 2 | 21 | 14 | 45 | 45 | 4 | 7 | 10 | 4 | 1 | 7 | 1 |
| 1 | X | 14 | 45 | 45 | 4 | 7 | 10 | 4 | 1 | 7 | 1 |
| 1 | X | 14 | 583 | 45 | 4 | 7 | 10 | 4 | 42 | 51 | 1 |
| 2 | 2 | 14 | 45 | 45 | 4 | 7 | 10 | 4 | 1 | 7 | 1 |
| 2 | 8 | 44 | 45 | 45 | 4 | 7 | 10 | 4 | 1 | 7 | 1 |
| 1 | 1235 | 971 | 2537 | 177 | 17 | 2 | 8 | 5 | 8 | 2 | 143 |
| 1 | 1170 | 132 | 2026 | 403 | 10 | 1 | 16 | 19 | 10 | 5 | 7 |
| 1 | 571 | 6 | 991 | 692 | 37 | 52 | 57 | 26 | 107 | 29 | 23 |
| 1 | 89 | 6 | 699 | 692 | 37 | 52 | 57 | 26 | 129 | 29 | 23 |
| 1 | 1067 | 6 | 692 | 692 | 37 | 52 | 57 | 26 | 127 | 29 | 23 |
| 2 | 209 | 180 | 4502 | 692 | 37 | 52 | 21 | 388 | 127 | 29 | 23 |
| 1 | X | X | 692 | 692 | 37 | 52 | 57 | 26 | 127 | 29 | 23 |
| 1 | 1067 | 152 | 2378 | 1034 | 2 | 15 | 4 | 48 | 356 | 25 | 23 |
| 1 | 16 | 792 | 1033 | 1034 | 2 | 61 | 4 | 64 | 126 | 7 | 23 |
| 4 | 56 | 879 | 2391 | 1034 | 2 | 15 | 4 | 48 | 360 | 25 | 23 |
| 4 | 22 | 973 | 1033 | 1034 | 2 | 61 | 4 | 64 | 126 | 7 | 23 |
| 2 | 1222 | 975 | 1255 | 1034 | 22 | 146 | 4 | 64 | 74 | 25 | 23 |
| 1 | X | X | 1033 | 1034 | 2 | 61 | 4 | 64 | 126 | 7 | 23 |
| 1 | 15 | 6 | 977 | 1034 | 22 | 61 | 4 | 64 | 74 | 25 | 23 |
| 1 | 89 | 6 | 977 | 1034 | 22 | 61 | 4 | 64 | 74 | 25 | 23 |
| 2 | X | X | 977 | 1034 | 22 | 61 | 4 | 64 | 74 | 25 | 23 |
| 1 | 1635 | X | 2378 | 1034 | 2 | 15 | 4 | 48 | 356 | 25 | 23 |
| 1 | 15 | 6 | 696 | 1332 | 2 | 1 | 4 | 28 | 58 | 25 | 58 |
| 1 | 56 | 886 | 696 | 1332 | 2 | 1 | 4 | 28 | 58 | 25 | 58 |
| 1 | 56 | 180 | 5648 | 692 | 37 | 418 | 57 | 26 | 129 | 29 | 23 |
| 1 | 1067 | 152 | 4496 | U/A | 2 | 15 | 98 | 48 | 356 | 25 | 23 |
| 1 | 73 | 879 | 4497 | U/A | 2 | 4 | 4 | 48 | 358 | 25 | 280 |
| 1 | 56 | 886 | 3961 | U/A | 2 | 29 | 4 | 27 | 10 | 25 | 24 |
| 1 | 1219 | 209 | 2354 | U/A | 37 | 4 | 4 | 48 | 13 | 25 | 23 |
| 2 | 69 | 888 | 710 | U/A | 37 | 29 | 75 | 48 | 126 | 25 | 23 |
| 1 | 787 | 180 | 2349 | U/A | 2 | 59 | 4 | 48 | 131 | 24 | 57 |
| 2 | 1221 | 888 | 2347 | U/A | 2 | 4 | 4 | 105 | 10 | 25 | 57 |
| 1 | X | 188 | 693 | U/A | 2 | 29 | 4 | 48 | 13 | 24 | 57 |
| 1 | 520 | 188 | 4500 | U/A | 2 | 29 | 296 | 48 | 131 | 25 | 57 |
| 1 | 213 | 819 | 693 | U/A | 2 | 29 | 4 | 48 | 13 | 24 | 57 |
| 1 | 1427 | X | 4501 | U/A | 237 | 2 | 254 | 340 | 433 | 349 | 290 |
| 1 | 219 | 188 | 992 | U/A | 2 | 59 | 4 | 27 | 126 | 29 | 23 |
| 1 | 1236 | 974 | 1324 | U/A | 99 | 128 | 91 | 125 | 170 | 146 | 111 |
| 1 | 22 | 973 | 2354 | U/A | 37 | 4 | 4 | 48 | 13 | 25 | 23 |
| 1 | 21 | 44 | 1342 | U/A | 98 | 122 | 98 | 125 | 180 | 150 | 113 |
| 1 | X | 152 | 995 | U/A | 2 | 4 | 84 | 105 | 126 | 25 | 57 |
| 1 | X | X | 4504 | U/A | 2 | 15 | 4 | 48 | 356 | 150 | 23 |
| 1 | 15 | 6 | 3961 | U/A | 2 | 29 | 4 | 27 | 10 | 25 | 24 |
| 1 | X | 152 | 995 | U/A | 2 | 4 | 84 | 105 | 126 | 25 | 57 |

X: Allele could not be typed

U/A: Unassigned

Table ST 2 – MLST allelic profiles, *fla*A, *por*A nucleotide alleles and their frequency in starlings.

| Frequency | *fla*A | *por*A | ST | CC | *asp*A | *gln*A | *glt*A | *gly*A | *pgm* | *tkt* | *unc*A |
| --- | --- | --- | --- | --- | --- | --- | --- | --- | --- | --- | --- |
| 1 | 321 | 180 | 5648 | 692 | 37 | 418 | 57 | 26 | 129 | 29 | 23 |
| 2 | 1067 | 152 | 2378 | 1034 | 2 | 15 | 4 | 48 | 356 | 25 | 23 |
| 1 | 1060 | 632 | 1304 | 1304 | 100 | 142 | 93 | 135 | 190 | 145 | 81 |
| 1 | X | X | 696 | 1332 | 2 | 1 | 4 | 28 | 58 | 25 | 58 |
| 3 | 322 | 971 | 177 | 177 | 17 | 2 | 8 | 5 | 8 | 2 | 4 |
| 1 | X | X | 177 | 177 | 17 | 2 | 8 | 5 | 8 | 2 | 4 |
| 1 | 341 | 236 | 177 | 177 | 17 | 2 | 8 | 5 | 8 | 2 | 4 |
| 1 | X | X | 53 | 21 | 2 | 1 | 21 | 3 | 2 | 1 | 5 |
| 1 | 1354 | 1074 | 4498 | 42 | 1 | 2 | 3 | 4 | 5 | 9 | 21 |
| 1 | 239 | 71 | 42 | 42 | 1 | 2 | 3 | 4 | 5 | 9 | 3 |
| 1 | 1237 | 970 | 45 | 45 | 4 | 7 | 10 | 4 | 1 | 7 | 1 |
| 1 | 21 | 14 | 45 | 45 | 4 | 7 | 10 | 4 | 1 | 7 | 1 |
| 5 | X | X | 45 | 45 | 4 | 7 | 10 | 4 | 1 | 7 | 1 |
| 1 | 1421 | 1185 | 583 | 45 | 4 | 7 | 10 | 4 | 42 | 51 | 1 |
| 2 | 2 | 44 | 45 | 45 | 4 | 7 | 10 | 4 | 1 | 7 | 1 |
| 1 | 239 | 73 | 583 | 45 | 4 | 7 | 10 | 4 | 42 | 51 | 1 |
| 1 | 21 | 44 | 45 | 45 | 4 | 7 | 10 | 4 | 1 | 7 | 1 |
| 1 | 8 | 44 | 45 | 45 | 4 | 7 | 10 | 4 | 1 | 7 | 1 |
| 1 | X | X | 137 | 45 | 4 | 7 | 10 | 4 | 42 | 7 | 1 |
| 1 | 321 | 53 | 137 | 45 | 4 | 7 | 10 | 4 | 42 | 7 | 1 |
| 1 | 1237 | 276 | 137 | 45 | 4 | 7 | 10 | 4 | 42 | 7 | 1 |
| 2 | X | X | 677 | 677 | 10 | 81 | 50 | 99 | 120 | 76 | 52 |
| 1 | 405 | 203 | 681 | 682 | 35 | 43 | 9 | 5 | 8 | 46 | 21 |
| 1 | X | 203 | 208 | 682 | 26 | 2 | 9 | 51 | 8 | 46 | 5 |
| 1 | 571 | 6 | 991 | 692 | 37 | 52 | 57 | 26 | 107 | 29 | 23 |
| 1 | 22 | 6 | 692 | 692 | 37 | 52 | 57 | 26 | 127 | 29 | 23 |
| 1 | X | X | 1342 | U/A | 98 | 122 | 98 | 125 | 180 | 150 | 113 |
| 1 | 21 | 14 | 992 | U/A | 2 | 59 | 4 | 27 | 126 | 29 | 23 |
| 1 | X | X | 3961 | U/A | 2 | 29 | 4 | 27 | 10 | 25 | 24 |
| 6 | 1237 | 970 | 4499 | U/A | 98 | 359 | 98 | 125 | 180 | 150 | 113 |
| 5 | 1236 | 974 | 1324 | U/A | 99 | 128 | 91 | 125 | 170 | 146 | 111 |
| 2 | 1237 | 970 | 1342 | U/A | 98 | 122 | 98 | 125 | 180 | 150 | 113 |
| 2 | 1236 | 972 | 1324 | U/A | 99 | 128 | 91 | 125 | 170 | 146 | 111 |
| 1 | 491 | NEW | 1286 | U/A | 95 | 2 | 94 | 127 | 172 | 144 | 114 |
| 1 | X | X | 2538 | U/A | 35 | 2 | 8 | 51 | 361 | 2 | 21 |
| 1 | 1421 | 1186 | 526 | U/A | 2 | 15 | 4 | 27 | 13 | 80 | 23 |
| 1 | X | X | 1324 | U/A | 99 | 128 | 91 | 125 | 170 | 146 | 111 |

X: Allele could not be typed

U/A: Unassigned

Table ST 3 –Clonal complexes, sequence types and their prevalence during summer in ducks (The months of spring and summer; September 2008 to February 2009 were categorised as summer as these months are warmer during the year):

| Prevalence (CC) | Frequency (CC) | CC | ST | Date | Frequency (ST) |
| --- | --- | --- | --- | --- | --- |
| 3.4 | 1 | 42 | 4503 | Sep-08 | 1 |
| 3.4 | 1 | 177 | 2537 | Sep-08 | 1 |
| 3.4 | 1 | 403 | 2026 | Sep-08 | 1 |
| 6.8 | 2 | 692 | 991 | Jan-09 | 1 |
|  |  |  | 5648 | Jan-09 | 1 |
| 27.6 | 8 | 1034 | 2391 | Feb-09 | 2 |
|  |  |  | 1033 | Nov-08 | 2 |
|  |  |  | 1033 | Dec-08 | 2 |
|  |  |  | 2378 | Feb-09 | 2 |
| 24.1 | 7 | 45 | 45 | Sep-08 | 2 |
|  |  |  | 45 | Oct-08 | 1 |
|  |  |  | 45 | Nov-08 | 2 |
|  |  |  | 45 | Dec-08 | 1 |
|  |  |  | 583 | Oct-08 | 1 |
| 3.4 | 1 | 692 | 699 | Feb-09 | 1 |
| 31.0 | 9 | U/A | 3961 | Jan-09 | 1 |
|  |  |  | 1342 | Sep-08 | 1 |
|  |  |  | 2354 | Sep-08 | 1 |
|  |  |  | 4496 | Feb-09 | 1 |
|  |  |  | 4497 | Feb-09 | 1 |
|  |  |  | 4504 | Jan-09 | 1 |
|  |  |  | 995 | Oct-08 | 2 |
| Total | 29 |  |  |  | 29 |

Table ST 4 –Clonal complexes, sequence types and their prevalence during winter in ducks (The months of autumn and winter; March to July 2009 and August 2008 were categorised as winter as these months are cooler during the year):

| Prevalence (CC) | Frequency (CC) | CC | ST | Date | Frequency (ST) |
| --- | --- | --- | --- | --- | --- |
| 5.7 | 2 | 1332 | 696 | Aug-08 | 2 |
| 28.6 | 10 | 1034 | 1033 | Mar-09 | 1 |
|  |  |  | 1033 | Jul-09 | 2 |
|  |  |  | 1255 | Jul-09 | 2 |
|  |  |  | 2378 | Jul-09 | 1 |
|  |  |  | 2391 | May-09 | 4 |
| 2.9 | 1 | 21 | 53 | Jun-09 | 1 |
| 14.3 | 5 | 45 | 583 | Apr-09 | 1 |
|  |  |  | 137 | May-09 | 1 |
|  |  |  | 45 | Jul-09 | 3 |
| 11.4 | 4 | 692 | 4502 | Jul-09 | 2 |
|  |  |  | 692 | May-09 | 1 |
|  |  |  | 699 | Jul-09 | 1 |
| 37.1 | 13 | U/A | 1324 | Aug-08 | 1 |
|  |  |  | 2347 | May-09 | 2 |
|  |  |  | 2349 | May-09 | 1 |
|  |  |  | 2354 | Mar-09 | 1 |
|  |  |  | 3961 | Mar-09 | 1 |
|  |  |  | 4500 | Jun-09 | 1 |
|  |  |  | 4501 | Jul-09 | 1 |
|  |  |  | 693 | Jun-09 | 2 |
|  |  |  | 710 | Apr-09 | 1 |
|  |  |  | 992 | Jun-09 | 1 |
|  |  |  | 992 | Jul-09 | 1 |
| Total | 35 |  |  |  | 35 |

Table ST 5 –Clonal complexes, sequence types and their prevalence during summer in starlings:

| Prevalence (CC) | Frequency (CC) | CC | ST | Date | Frequency (ST) |
| --- | --- | --- | --- | --- | --- |
| 7.1 | 2 | 1034 | 2378 | Feb-09 | 2 |
| 3.6 | 1 | 1304 | 1304 | Feb-09 | 1 |
| 3.6 | 1 | 1332 | 696 | Oct-08 | 1 |
| 7.1 | 2 | 177 | 177 | Oct-08 | 2 |
| 46.4 | 13 | 45 | 137 | Nov-08 | 3 |
|  |  |  | 45 | Sep-08 | 2 |
|  |  |  | 45 | Oct-08 | 2 |
|  |  |  | 45 | Nov-08 | 1 |
|  |  |  | 45 | Jan-09 | 1 |
|  |  |  | 45 | Feb-09 | 1 |
|  |  |  | 583 | Oct-08 | 1 |
|  |  |  | 583 | Nov-08 | 1 |
|  |  |  | 583 | Dec-08 | 1 |
| 7.1 | 2 | 677 | 677 | Nov-08 | 2 |
| 25.0 | 7 | U/A | 1324 | Oct-08 | 1 |
|  |  |  | 1342 | Jan-09 | 1 |
|  |  |  | 3961 | Feb-09 | 1 |
|  |  |  | 4499 | Dec-08 | 3 |
|  |  |  | 992 | Feb-09 | 1 |
| Total | 28 |  |  |  | 28 |

Table ST 6 –Clonal complexes, sequence types and their prevalence during winter in starlings:

| Prevalence (CC) | Frequency (CC) | CC | ST | Date | Frequency (ST) |
| --- | --- | --- | --- | --- | --- |
| 10.3 | 3 | 177 | 177 | Apr-09 | 2 |
|  |  |  | 177 | Jun-09 | 1 |
| 3.4 | 1 | 21 | 53 | Jul-09 | 1 |
| 6.9 | 2 | 42 | 42 | Jul-09 | 1 |
|  |  |  | 4498 | Mar-09 | 1 |
| 10.3 | 3 | 45 | 45 | Jun-09 | 2 |
|  |  |  | 583 | Aug-08 | 1 |
| 6.9 | 2 | 682 | 208 | Jun-09 | 1 |
|  |  |  | 681 | Apr-09 | 1 |
| 10.3 | 3 | 692 | 692 | Jun-09 | 1 |
|  |  |  | 991 | Mar-09 | 1 |
|  |  |  | 5648 | Aug-08 | 1 |
| 55.2 | 16 | U/A | 1286 | Jul-09 | 1 |
|  |  |  | 1324 | Apr-09 | 1 |
|  |  |  | 1324 | May-09 | 3 |
|  |  |  | 1324 | Jun-09 | 2 |
|  |  |  | 1324 | Jul-09 | 1 |
|  |  |  | 1324 | Apr-09 | 2 |
|  |  |  | 2538 | Aug-08 | 1 |
|  |  |  | 4499 | Mar-09 | 1 |
|  |  |  | 4499 | Jul-09 | 2 |
|  |  |  | 526 | Aug-08 | 1 |
| Total | 29 |  |  |  | 29 |

Table ST 7: Analysis of molecular variance (AMOVA)

Population pairwise Fsts for ducks and starlings at different sampling sites

|  | ESS | HKS | MSS | MMS | SQS | ESD | HKD | MSD | MMD | SQD |
| --- | --- | --- | --- | --- | --- | --- | --- | --- | --- | --- |
| ESS | 0 | 0 | 0.063 | 0.117 | **0.009** | **0** | 0.036 | **0** | 0.036 | **0** |
| HKS | 0.224 | 0 | 0.072 | **0.009** | 0.135 | **0** | 0.396 | **0.009** | 0.072 | **0.018** |
| MSS | 0.226 | 0.22 | 0 | 0.162 | 0.261 | **0** | 0.144 | **0** | 0.108 | **0.027** |
| MMS | 0.075 | 0.156 | 0.069 | 0 | 0.135 | **0.009** | 0.216 | 0.063 | 0.811 | **0.009** |
| SQS | 0.162 | 0.052 | 0.048 | 0.053 | 0 | **0.018** | 0.261 | 0.153 | 0.333 | 0.144 |
| ESD | 0.276 | 0.187 | 0.292 | 0.172 | 0.06 | 0 | 0.09 | 0.396 | 0.009 | 0.775 |
| HKD | 0.203 | 0.014 | 0.135 | 0.06 | 0.015 | 0.069 | 0 | 0.144 | 0.748 | **0.045** |
| MSD | 0.248 | 0.208 | 0.321 | 0.15 | 0.043 | -0.003 | 0.078 | 0 | 0.18 | 0.351 |
| MMD | 0.107 | 0.083 | 0.069 | -0.051 | 0.005 | 0.086 | -0.046 | 0.058 | 0 | **0.009** |
| SQD | 0.270 | 0.132 | 0.267 | 0.173 | 0.047 | -0.015 | 0.061 | 0.007 | 0.087 | 0 |

Note: The values below the diagonal are the Fst values and the values above the diagonal are the p-values. The significant values are shown in bold fonts.

Populations compared:

ESS : Esplanade starlings; HKS : Hokowhitu starlings ; MSS : Massey starlings ; MMS : Memorial starlings ; SQS : The Square starlings ;

ESD : Esplanade ducks ; HKD : Hokowhitu ducks ; MSD : Massey ducks ; MMD : Memorial ducks ; SQD : The Square ducks.

**Cell surface antigen typing:**

The isolates that were difficult to amplify were further processed in the Hopkirk Research Laboratory using primers from the PubMLST database. The PCR program was optimised to amplify the difficult *fla*A and *por*A sequences and further, the MgCl2 was optimised to 2.5 M and primers to 3.2 picomoles. The modified PCR program used for amplifying and sequencing the difficult isolates is described in the Table ST8.

Table ST 8: *fla*A and *por*A typing

| *por*A PCR reaction and program: | *fla*A PCR reaction and program: |
| --- | --- |
| Primers: Momp1 and Momp2 from the PUBMLST database: | Primers: *fla*A1 and *fla*A2 from the PUBMLST database: |
| 10X buffer: 2 µL | 10X buffer: 2 µL |
| MgCl2 - 1 µL | MgCl2 - 1 µL |
| DNTPs - 1 µL | DNTPs - 1 µL |
| Primer1 - 2 µL | Primer1 - 2 µL |
| Primer2 - 2 µL | Primer2 - 2 µL |
| Taq(Platinum) - 0.2 µL | Taq(Platinum) - 0.2 µL |
| DNA (50 - 100ng) - 2 µL | DNA (50 - 100ng) - 2 µL |
| Made upto 20µL with distilled water | Made upto 20µL with distilled water |
|  |  |
| PCR program: | PCR program: |
| Initial denaturation 95°C for 2 minutes for 40 cycles: | Initial denaturation 94°C for 2 minutes for 37 cycles: |
| Denaturation 94 °C: 30 seconds | Denaturation 94 °C: 30 seconds |
| Annealing 50 °C: 30 seconds | Annealing 64 °C: 30 seconds |
| Extension 72 °C: 30 seconds | Extension 72 °C: 45 seconds |
| Final extension: 72 °C for 2 minutes | Final extension: 72 °C for 2 minutes |
